# Numerical Study on the Characteristics of Boger Type Viscoelastic Fluid Flow in a Micro Cross-Slot under Sinusoidal Stimulation

**DOI:** 10.3390/e22010064

**Published:** 2020-01-03

**Authors:** Chao Yuan, Hong-Na Zhang, Li-Xia Chen, Jun-Long Zhao, Xiao-Bin Li, Feng-Chen Li

**Affiliations:** 1 School of Aeronautics and Astronautics, Sun Yat-sen University, Guangzhou 510006, China; yuanch9@mail2.sysu.edu.cn; 2 Sino-French Institute of Nuclear Engineering and Technology, Sun Yat-sen University, Zhuhai 519082, China; chenlx59@mail2.sysu.edu.cn (L.-X.C.); zhaojlong3@mail.sysu.edu.cn (J.-L.Z.); lixiaobin@mail.sysu.edu.cn (X.-B.L.)

**Keywords:** viscoelastic fluid, micro cross-slot channel, microfluidics, external stimulation

## Abstract

The cross-slot geometry plays an important role in the study of nonlinear effects of viscoelastic fluids. The flow of viscoelastic fluid in a micro cross-slot with a high channel aspect ratio (*AR*, the ratio of channel depth to width) can be divided into three types, which are symmetric flow, steady-state asymmetric flow and time-dependent flow under the inlet condition with a constant velocity. However, the flow pattern of a viscoelastic fluid in the cross-slot when a stimulation is applied at inlets has been rarely reported. In this paper, the response of cross-slot flow under an external sinusoidal stimulation is studied by numerical simulations of a two-dimensional model representing the geometry with a maximum limit of *AR*. For the cases under constant inlet velocity conditions, three different flow patterns occur successively with the increase of Weissenberg number (*Wi*). For the cases under sinusoidal varying inlet velocity conditions, when the stimulation frequency is far away from the natural frequency of a viscoelastic fluid, the frequency spectrum of velocity fluctuation field shows the characteristics of a fundamental frequency and several harmonics. However, the harmonic frequency disappears when the stimulation frequency is close to the natural frequency of the viscoelastic fluid. Besides, the flow pattern shows spatial symmetry and changes with time. In conclusion, the external stimulation has an effect on the flow pattern of viscoelastic fluid in the 2D micro cross-slot channel, and a resonance occurs when the stimulation frequency is close to the natural frequency of the fluid.

## 1. Introduction

As the technology of manipulation and control of microscale liquids becomes more and more mature, microfluidics gradually becomes a separate academic subject. It has been widely used in many fields, such as micro-mixer [1,2,3], micro heat exchanger [4,5], logic circuits [6,7], particle manipulation [8,9], liquid computers [10], and so forth. Working as one of the common medium in microfluidics, viscoelastic fluid can be generally divided into three groups, polymer solutions, surfactant solutions, and polymer melts. It has both the inertial and elastic nonlinearities, which can induce an elastic instability [11,12] and even elastic turbulence [13], thus resulting in many unique phenomena, like the shear thinning-/thickening [14], turbulent drag reduction [15], rod-climbing effect (Weissenberg effect) [16], tubeless siphon phenomenon [17], extrudate swelling effect [18], bouncing liquid jet effect (Kaye Effect) [19], and so on.

Micro-scale cross-slot channel is one of the most basic channel structures and widely used in the study of the nonlinear effect of viscoelastic fluids. Due to the strong tensile effect of cross-slot channel at the junction of channels, it is often used in the measurement of rheological tensile viscosity [20].

Research on viscoelastic fluid flow in cross-slot channel was started by experiments. Arratia et al. [21] first observed that there exist three flow patterns of dilute polymer solution in a cross-slot channel. The flow patterns include the following; (1) symmetric flow, at the Weissenberg number (*Wi*) smaller than the 1st critical *Wi* (*Wi_c_*_1_), i.e., *Wi* < *Wi_c_*_1_; (2) steady asymmetric flow, at *Wi_c_*_1_ < *Wi* < *Wi_c_*_2_; (3) time-dependent flow, at *Wi* > *Wi_c_*_2_. Pure elastic instability flow includes the latter two flow patterns, and the random flow phenomenon of the third unsteady flow pattern is similar to inertial turbulence, so it is also called elastic turbulence [22]. Pathak and Hudson [23] also observed the above three flow patterns by using micelle solution as the working fluid in a cross-slot channel. Further studies by Sousa et al. [24] showed that the three flow patterns do not appear successively with the increase of *Wi*, but are related to the physical properties of the working fluid itself and the channel aspect ratio *AR*, which is defined as the ratio of channel depth to width. Subsequent studies had been carried out by means of numerical simulation. Cruz et al. [25] proposed stationary bifurcation in a cross-slot as a new viscoelastic benchmark flow. Rocha et al. [26] used FENE-CR and FENE-P constitutive models to simulate the rheological properties of constant shear viscosity and shear-thinning fluid in cross-slot channel, respectively. Afonso et al. [27] studied the effect of external stimulation on the flow dynamics in the cross-slot channel with a main inlet flow and two lateral inlets at opposing directions. They aimed at the potential application of viscoelastic fluid to the flow-focusing fields, and found that the flow dynamics at outlet channel is dependent on the frequency, amplitude, and phase of the external stimulation applied at the two lateral inlets.

This paper aims at the effect of external stimulation on the flow dynamics in the cross-slot channel with two opposing inlets and two opposing outlets. The rest of the paper is organized as follows. In Section 2, the numerical methods are described in detail. The results and discussion are presented in Section 3. In this section, the effect of external stimulation on flow dynamics is analyzed from the following aspects, i.e., flow patterns, time domain, and frequency domain analysis. The conclusions are finally drawn in Section 4.

## 2. Methods

### 2.1. Physical Model

**Figure 1:**
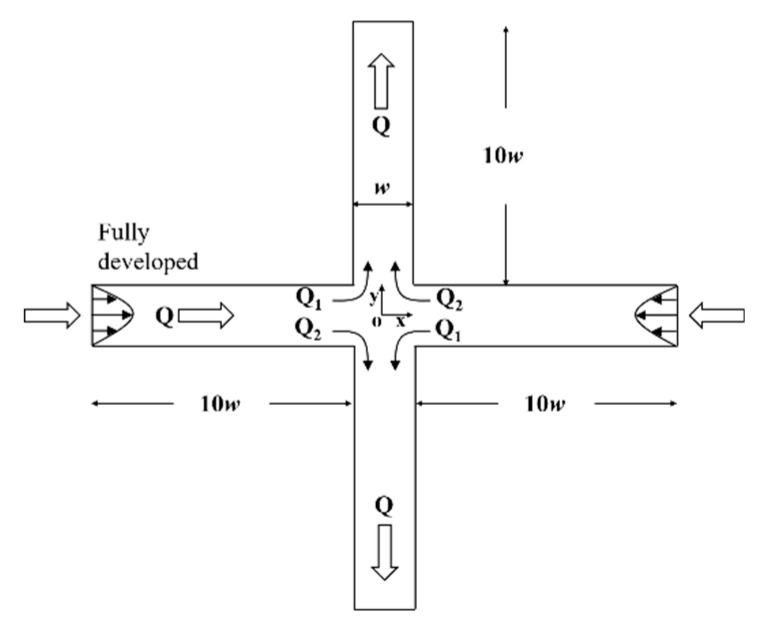
Schematic of the 2D cross-slot channel.

The structure of the two-dimensional (2D) cross-slot channel is shown in Figure 1. The left and right sides of the channel are the entrances in opposite directions, and the upper and lower sides are the exits. The width of the channel is *w* and the length of the four branches is set as 10*w*. The origin is located at the center of the cross channel, i.e., the stagnation point.

### 2.2. Governing Equations of Viscoelastic Fluid Flow

Under the assumption of continuity and incompressibility, the conservation equation of continuity and momentum can be obtained. For the sake of generality, a set of dimensionless scales are introduced as follows,

(1) $$u^{+}=u/U,\;t^{+}=t\cdot U/w,\;p^{+}=p/\left(\rho \cdot U^{2}\right),\;\nabla^{+}=w\cdot \nabla$$

where, u is the velocity vector, U is the characteristic velocity, *t* is the time, *w* is the channel width, p is the pressure, *ρ* is the density, and ∇ is the Hamiltonian operator. The superscript + represents dimensionless variables. Then, the corresponding dimensionless governing equations can be written as follows,

(2) $$\nabla^{+}\cdot u^{+}=0$$

(3) $$\frac{\partial u^{+}}{\partial t^{+}}+u^{+}\cdot \nabla^{+}u^{+}=-\nabla^{+}p^{+}+\frac{\beta }{Re}\nabla^{+}{}^{2}u^{+}+\frac{1-\beta }{Re\cdot Wi}\nabla^{+}C^{+}$$

(4) $$\frac{\partial C^{+}}{\partial t^{+}}+u^{+}\cdot \nabla^{+}C^{+}=C^{+}\cdot \nabla^{+}u^{+}+{\left(\nabla^{+}u^{+}\right)}^{T}\cdot C^{+}+\frac{1}{Wi}\left[I+\alpha \left(C^{+}-I\right)\right]\cdot \left[f\left(r\right)C^{+}-I\right]$$

where, β is the ratio of solvent dynamic viscosity to the zero-shear viscosity, C is the conformation tensor, superscript ***T*** stands for transpose operator, α is the mobility factor, I is the unit tensor, and f(r) is the nonlinear stretching factor of viscoelastic fluid molecules. The specific values of α and f(r) corresponding to the commonly used Oldroyd-B, FENE-P and Giesekus constitutive models are given in [28]. The dimensionless parameters Re and Wi are the Reynolds number and Weissenberg number, which are defined as Re=(ρUw)/μ and Wi=(λU)/w, respectively, where, μ is the zero-shear viscosity and λ is the relaxation time.

### 2.3. Numerical Methods

To deal with the so-called “high Weissenberg number problem (HWNP)”, a logarithmic conformation reformulation (LCR) algorithm is adopted. Kupferman and Fattal et al. [29,30,31] proposed a simple logarithmic transformation of the deformation rate tensor, which is transformed from solving the multiplication form of the deformation rate tensor to solving the summation form of the exponent of the deformation rate tensor, so as to reduce the error of polynomial fitting to ensure the correctness and stability of calculation. This method has been proved to be effective [32]. For the discretization method, the Euler scheme is used for the transient term, the QUICK scheme for the velocity convection term and the Gauss scheme for the Laplacian term. A PISO algorithm is adopted to solve the coupling of pressure and velocity fields.

### 2.4. Boundary Conditions

**Figure 2:**
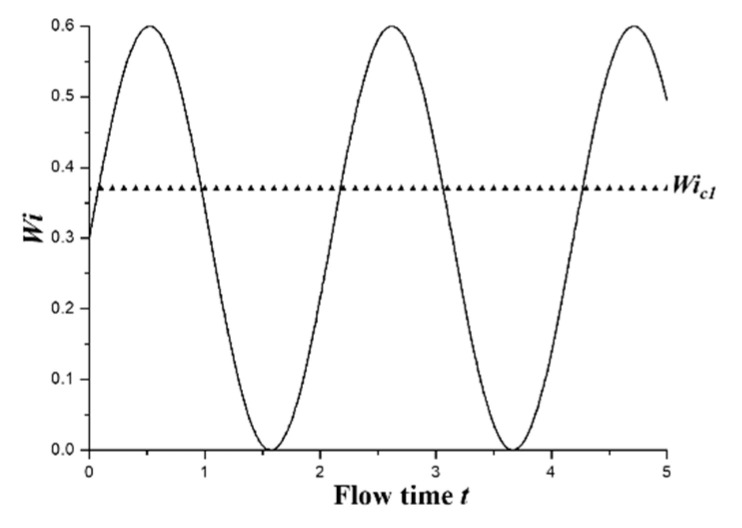
Variation of *Wi* with time *t*.

The boundary conditions (BCs) of a constant pressure of 0 Pa are set at the outlets. At the channel walls, the no-slip condition is considered. Two kinds of BCs are applied at the inlets: (1) Constant velocity: in this case, the flow of viscoelastic fluid with different *Wi* numbers can be obtained (to eliminate the influence of the entrance section, a fully developed velocity profile is set at the channel inlets). (2) Sinusoidal varying velocity: the inlet velocity changes sinusoidally with time and is defined as

(5) $$u=U+A\cdot \sin\left(2\pi f_{0}\cdot t\right))$$

where *u* is the instantaneous velocity component in X direction, *A* is the amplitude to measure the degree of pulsation of external stimulation and *f*_0_ is the external stimulation frequency. The parameters of the sinusoidal signal at the inlets are shown in Table 1. These parameters are specially chosen to make sure that the flow condition will cross the first critical *Wi* number *Wi_c_*_1_ periodically, as shown in Figure 2, so that the impact of different stimulations on the flow patterns can be investigated. To avoid misunderstanding, it is worthy to point out that *Wi* can be defined based on the time-averaged mean velocity U (which is *Wi_m_*) and the time-dependent mean velocity *u* (*Wi*), respectively, for the viscoelastic fluid flow under an external stimulation. Therefore, the value of *Wi_m_* is 0.3 for different external stimulation frequencies.

To eliminate the effect of shear thinning on the results, Boger [33] type viscoelastic fluid with almost constant viscosity is chosen as the working fluid. The typical value of viscosity ratio *β* is 0.1, which represents a concentrated solution.

### 2.5. Grid Independence Validation

Structured grid is used for mesh generation. Six sets of meshes are obtained by increasing the number of cells in the transverse and longitudinal directions of the cross channel, the details of which are tabulated in Table 2. The measurement parameter of grid independence is the velocity modulus at (x, y) = (0, *w*).

**Figure 3:**
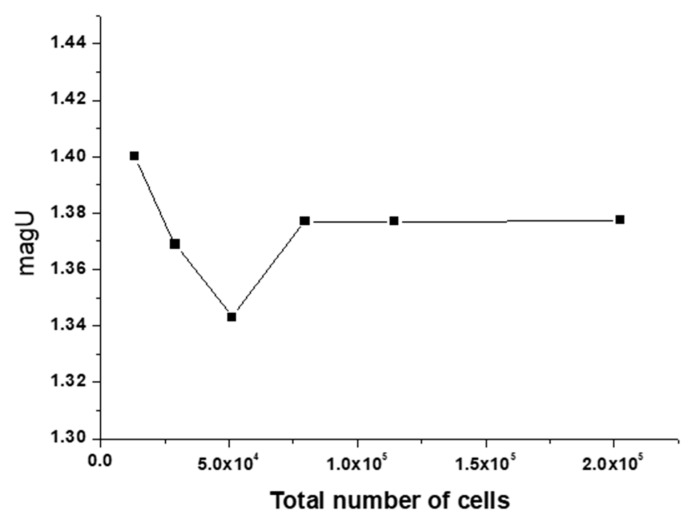
The velocity modulus at (0, *w*) as a function of total number of cells.

To verify that the grid independence is applicable to all three flow states, the case of constant inlet velocity with *Wi* = 2 is taken as a typical example to test the grid independence. Note that the flow at high *Wi* is time-dependent, so the selected parameters are statistically averaged over time. Figure 3 shows that the velocity modulus fluctuates with the number of increasing nodes, but tends to be stable value at the fourth set. The subsequent research is based on Mesh 4.

## 3. Results and Discussion

### 3.1. Flow Pattern and DQ

The typical elastic instability of a viscoelastic fluid in micro cross-slot channel is a bifurcation flow, and the largest *Re* for all the cases in this paper is approximately 0.2, which indicates that the elastic instability occurred in the cross-slot channel is induced mainly by the elastic effect of the viscoelastic fluids, i.e., purely elastic instability. Poole et al. [34] proposed a bifurcation parameter *DQ* to measure the degree of flow asymmetry, which is defined as

*DQ* = (*Q*_2_ − *Q*_1_)/(*Q*_2_ + *Q*_1_) (6)

where *Q*_1_ and *Q*_2_ represent the divided flow rates in one inlet channel flowing into two outlet channels, as depicted in Figure 1. The two limiting cases of *DQ* = 0 and ±1 represent the situations of symmetric flow and completely asymmetric flow, respectively.

#### 3.1.1. Constant Inlet Velocity Condition

**Figure 4:**
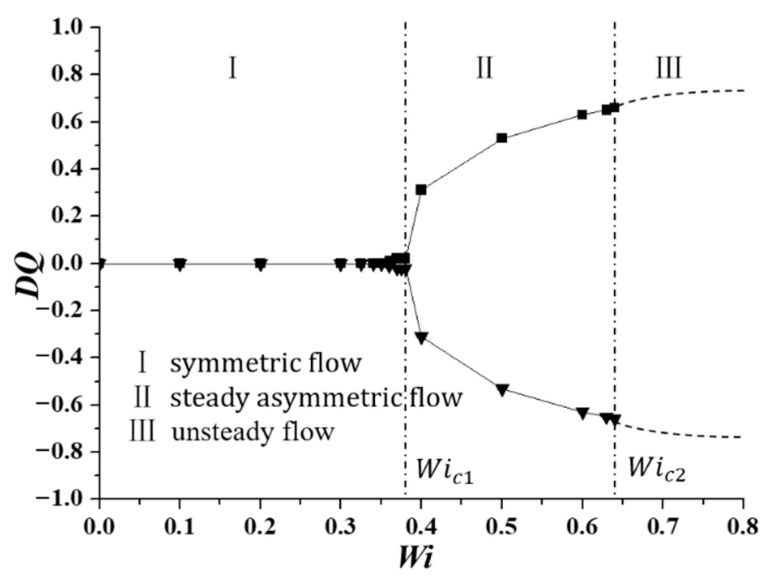
Curves of *DQ* as a function of *Wi* at constant inlet velocity condition.

**Figure 5:**
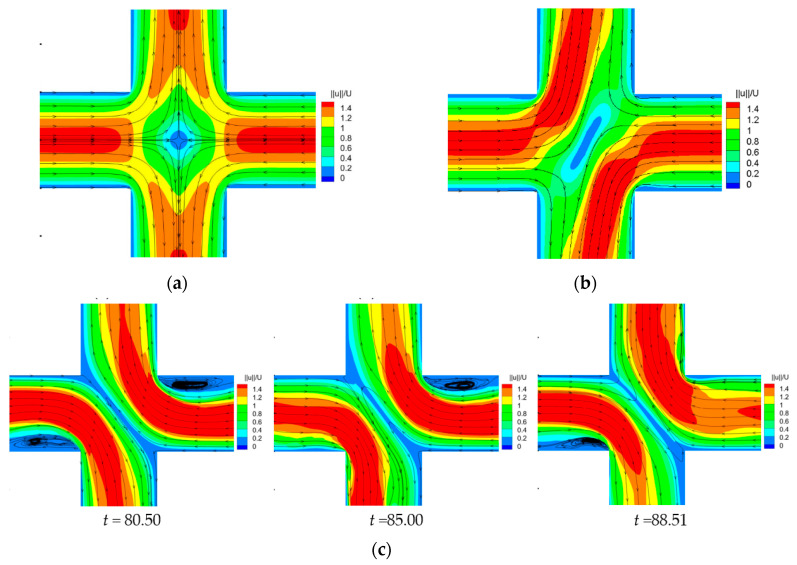
Velocity contours and superimposed streamlines at different *Wi* numbers. (**a**) *Wi* = 0.2. (**b**) *Wi* = 0.4. (**c**) *Wi* = 2 at different times.

The classical pitchfork bifurcation trend of *DQ* changing with *Wi* is plotted in Figure 4. Figure 5 shows the velocity contours and superimposed streamlines at typical *Wi* numbers in the corresponding flow regions. It can be seen that the change of *DQ* with *Wi* has three typical characteristics. (1) When *Wi* is small enough, the flow pattern is completely symmetric, and the value of *DQ* is 0. (2) With *Wi* increasing, the flow pattern changes from symmetric flow to steady asymmetric flow, and the first critical *Wi* number *Wi_c_*_1_ is 0.370 (square root fit, *DQ* $=B\sqrt{Wi-Wi_{cr1}}$, where *B* = 1.30), which is close to the result of 0.363 [25]. (3) With *Wi* further increasing, the flow pattern becomes time-dependent. The second critical *Wi* number *Wi_c_*_2_ is determined by the boundary and the flow in two sides of which are steady asymmetric state and time-dependent state. The value of *Wi_c_*_2_ is 0.645, which is slightly larger than the study of Cruz et al. [25], 0.425. Many different aspects may affect the difference of the results, such as the total number of mesh nodes, discretization method for different terms in governing equations, numerical method to solve the system of differential equations. Besides, the viscosity ratio in our study and theirs are 0.1 and 1/9, which may be another one of the reasons to explain the differences between the results.

#### 3.1.2. Sinusoidal Varying Inlet Velocity Condition

**Figure 6:**
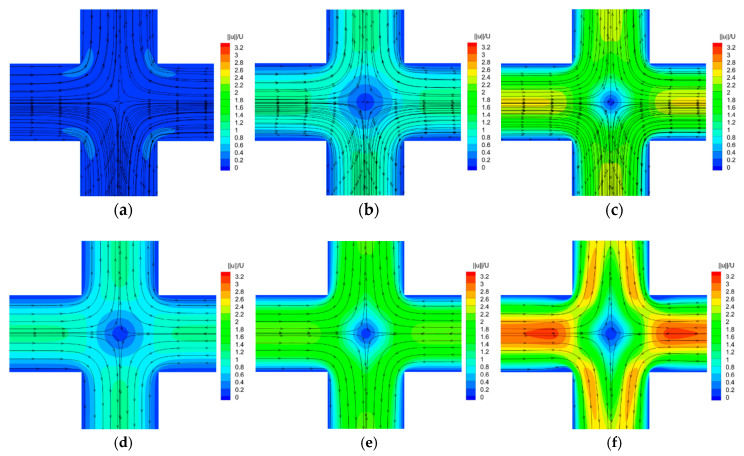
Velocity contours and superimposed streamlines at different stimulation frequency *f*_0_. Upper rows are for *f*_0_ = 0.149 Hz at different *Wi*. Lower rows are for *f*_0_ = 0.074 Hz at different *Wi*. (**a**) *f*_0_ = 0.149 Hz (*t* = 187.09; *Wi* = 0.1);.(**b**) *f*_0_ = 0.149 Hz (*t* = 187.92; *Wi* = 0.3); (**c**) *f*_0_ = 0.149 Hz (*t* = 188.91; *Wi* = 0.5); (**d**) *f*_0_ = 0.074 Hz (*t* = 187.52; *Wi* = 0.1); (**e**) *f*_0_ = 0.074 Hz (*t* = 189.19; *Wi* = 0.3); (**f**) *f*_0_ = 0.074 Hz (*t* = 191.18; *Wi* = 0.5).

When sinusoidal varying velocity is applied at the channel inlets, the *Wi* of viscoelastic fluid changes from nearly 0 to 0.6 in one external stimulation cycle, and the change range covers two flow regimes under the constant inlet velocity condition. At this time, the expected flow pattern may be the same as that without stimulation, i.e., two flow patterns may occur successively. However, as shown in Figure 6, the flow states are all symmetric at both different *Wi* numbers and external stimulation frequencies.

**Figure 7:**
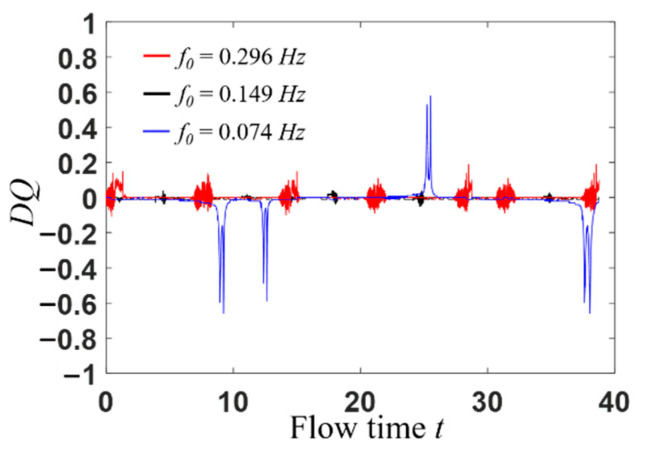
Curves of *DQ* as a function of time *t* under sinusoidal varying inlet velocity condition.

**Figure 8:**
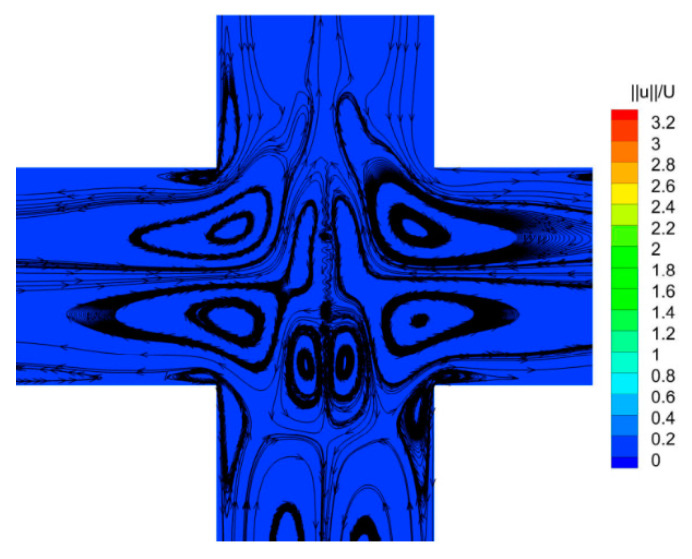
Velocity contours and superimposed streamlines in the vicinity of the stagnation point. *f*_0_ is 0.149 Hz and inlet velocity approaches 0.

From the quantitative point of view, the flow pattern of a viscoelastic fluid under the external sinusoidal stimulation is analyzed. From Figure 7, it can be seen that *DQ* is close to 0 at most of the times, which indicates the flow pattern is symmetric. However, as the inlet velocity approaches zero, the viscoelastic fluid molecule structures change from a strong tensile state to relaxed state, then a complicated flow appears in the vicinity of the stagnation point, as shown in Figure 8. In this case, the bifurcation parameter *DQ* is chaotic.

### 3.2. Time Domain Analysis

**Figure 9:**
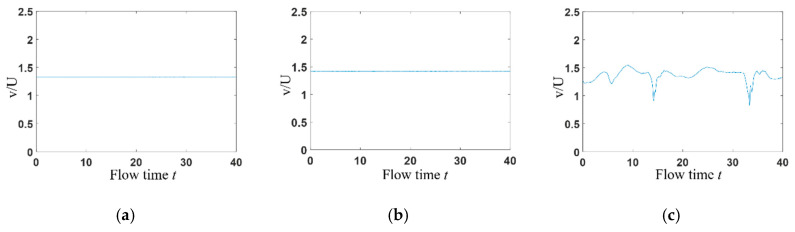
Time domain diagram of transient velocity at (x, y) = (0, *w*) without stimulation. (**a**) *Wi* = 0.3; (**b**) *Wi* = 0.4; (**c**) *Wi* = 2.

**Figure 10:**
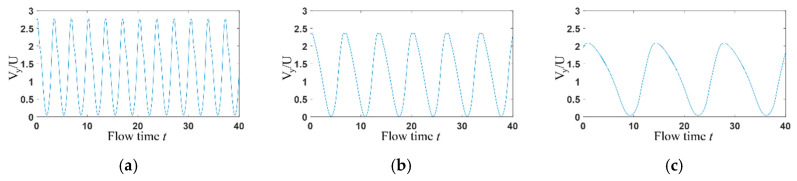
Time domain diagram of transient velocity v in Y direction at (x, y) = (0, *w*) with sinusoidal stimulation. (**a**) *f*_0_ = 0.296 Hz, *Wi_m_* = 0.3; (**b**) *f*_0_ = 0.149 Hz, *Wi_m_* = 0.3; (**c**) *f*_0_ = 0.074 Hz, *Wi_m_* = 0.3.

The transient velocity at (0, *w*) is selected for the statistical analysis of the flow field. In the microchannel, the inertial effect of the flow can be neglected. When the velocity is constant at inlets, it can be seen from Figure 9 that the flow is steady (steady symmetric flow and steady asymmetric flow) with *Wi* lower than the *Wi_c_*_2_, but shows a strong pulsation when elastic effect is strong enough (Figure 9c), indicating that elastic turbulence occurs at this time. However, when sinusoidal varying velocity is applied at channel inlets, Figure 10 presents that velocity at (x, y) = (0, *w*) changes sinusoidally with time.

### 3.3. Frequency Domain Analysis

**Figure 11:**
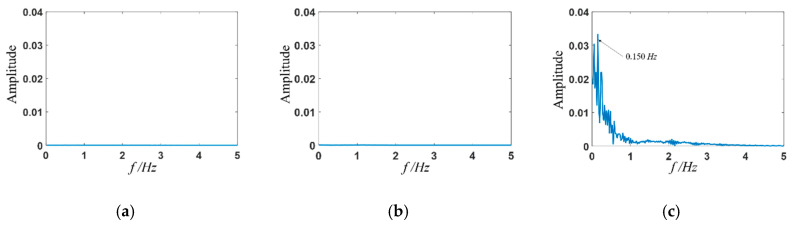
Amplitude spectrum of transient velocity at (x, y) = (0, *w*) without stimulation. (**a**) *Wi* = 0.3; (**b**) *Wi* = 0.4; (**c**) *Wi* = 2.

**Figure 12:**
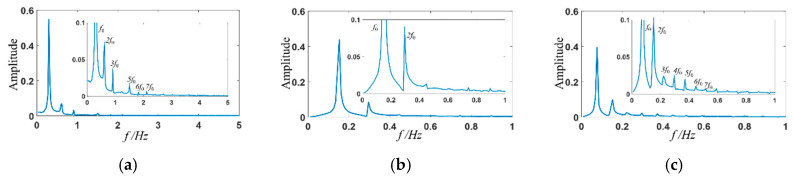
Amplitude spectrum of transient velocity at (x, y) = (0, *w*) with sinusoidal stimulation. (**a**) *f*_0_ = 0.296 Hz; (**b**) *f*_0_ = 0.149 Hz; (**c**) *f*_0_ = 0.074 Hz.

The viscoelastic fluid flow at high *Wi* has a random elastic turbulent motion. More detailed information can be obtained from the spectrum analysis, as shown in Figure 11 and Figure 12. For the case of constant inlet velocity at channel inlets, the viscoelastic fluid flow states are steady with *Wi* smaller than *Wi_c_*_2._ As *Wi* increases further, a main frequency of viscoelastic fluid occurs and the value of which is 0.150 Hz corresponding to *Wi* = 2 (Figure 11c). When the sinusoidal varying velocity is applied, different results are presented according to the relationship between the stimulation frequency and the natural frequency of viscoelastic fluid: (1) When the stimulation frequency is far away from the natural frequency, like the stimulation frequency *f*_0_ = 0.296 (Figure 12a) and *f*_0_ = 0.074 Hz (Figure 12c), the frequency spectrum presents one fundamental frequency and multiple harmonic frequencies. (2) When the stimulation frequency approaches to the natural frequency, like the stimulation frequency *f*_0_ = 0.149 Hz (Figure 12b), a resonance phenomenon occurs and is characterized by only one fundamental frequency and disappearance of most harmonic frequencies. Herein, the natural frequency of viscoelastic fluid with *Wi* in the range of 0 to 0.6 is around 0.149 Hz.

**Figure 13:**
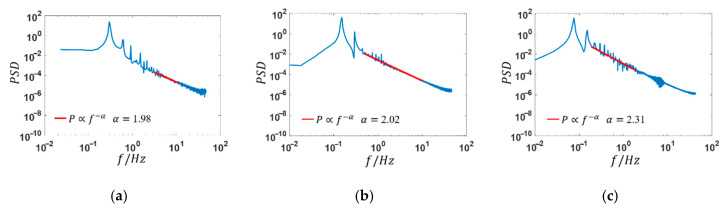
Power spectrum of transient velocity at (x, y) = (0, *w*) with sinusoidal stimulation. (**a**) *f*_0_ = 0.296 Hz; (**b**) *f*_0_ = 0.149 Hz; (**c**) *f*_0_ = 0.074 Hz.

The power spectrum density (PSD) of instantaneous velocity, as shown in Figure 13, satisfies the law of exponential decay ($P\propto f^{-\alpha }$) in at least two orders of magnitude with the change of frequency, and the decay exponents are −2.31, −2.02, and −1.98 corresponding to different external stimulation frequencies. The results are qualitatively consistent with the findings obtained by Sousa et al. [35], whose studies showed that the velocity power spectra exponent of elastic turbulence in a microfluidic cross-slot device is between −2 and −4.

For the constant inlet condition, the steady asymmetric flow pattern can be formed in a certain time. However, when external stimulation is applied, the viscoelastic fluid has not enough time to transfer to the steady-state asymmetric flow pattern but remain a steady state at most of the time, which may be the reason for the appearance of symmetric pattern in space under the external in-phase stimulation. Note that the above results are obtained with relatively lower amplitude of sinusoidal varying *Wi* and in-phase external stimulation configuration. As the amplitude of varying *Wi* progressively increases and the in-phase configuration changes to out-of-phase, the results may be much different from this work.

## 4. Conclusions

For concentrated viscoelastic fluid flowing in a 2D micro cross-slot channel, as *Wi* increases, three flow patterns occur successively, i.e., symmetric flow, steady asymmetric flow, and time-dependent flow. In this paper, the response of viscoelastic fluid to external sinusoidal varying stimulation signal is studied by numerical simulation. The conclusions are as follows.

(1) From the view of bifurcation parameter *DQ*, the flow pattern in micro cross-slot is time-dependent, but remains symmetric in space.
(2) When the stimulation frequency is far away from the natural frequency of the viscoelastic fluid, the frequency spectrum of velocity fluctuation field shows the characteristics of a fundamental frequency and several harmonics. However, when the stimulation frequency is close to the natural frequency, a resonance phenomenon occurs, characterized by only one fundamental frequency and the disappearance of most harmonic frequencies.
(3) The decay exponent of PSD of instantaneous velocity is between −2.31 and −1.98.

## Figures and Tables

**Table 1:** Parameter settings of sinusoidal varying velocity at inlets.

*f*_0_	*U*	*A*	*λ*
0.296	1	1	0.3
0.149	1	1	0.3
0.074	1	1	0.3

**Table 2:** Characteristics of the meshes used in simulations.

Item	ND_x_	ND_y_	ND_branch_	NC	ND
Mesh1	51	51	51	13,112	12,801
Mesh2	76	76	76	29,037	28,576
Mesh3	101	101	101	51,212	50,601
Mesh4	126	126	126	79,637	78,876
Mesh5	151	151	151	114,312	113,401
Mesh6	201	201	201	202,412	201,201

Note: ND_x_ and ND_y_ are the number of nodes in the X and Y direction of the central region, respectively. ND_branch_ is the number of nodes along the four branches. NC and ND are the total number of cells and nodes, respectively.
